# Nutritional composition of *Eragrostis teff* and its association with the observed antimutagenic effects

**DOI:** 10.1039/c8ra09733j

**Published:** 2019-01-28

**Authors:** Maria Clara da Silva Goersch, Laura Schäfer, Marina Tonial, Viviani Ruffo de Oliveira, Alexandre de Barros Falcão Ferraz, Jean Fachini, Juliana Bondan da Silva, Liana Appel Boufleur Niekraszewicz, Carlos Eduardo Rodrigues, Giancarlo Pasquali, Johnny Ferraz Dias, Tarso B. Ledur Kist, Jaqueline Nascimento Picada

**Affiliations:** Graduating Program in Cell and Molecular Biology Applied to Health, Laboratory of Toxicological Genetics, Lutheran University of Brazil (ULBRA) Farroupilha Avenue 8001 92425-900 Canoas RS Brazil jnpicada@gmail.com +55 51 34771313 +55 51 34779158; Laboratory of Methods, Department of Biophysics, Institute of Biosciences, Federal University of Rio Grande do Sul Bento Goncalves Avenue 9500 Porto Alegre RS Brazil; Department of Nutrition, Medical School, Federal University of Rio Grande do Sul Ramiro Barcelos Street 2400 Porto Alegre RS Brazil; Laboratory of Phytochemistry, Lutheran University of Brazil (ULBRA) Farroupilha Avenue 8001 Canoas RS Brazil; Ion Implantation Laboratory (LII), Institute of Physics, Federal University of Rio Grande do Sul Bento Goncalves Avenue 9500 Porto Alegre RS Brazil; Graduating Program in Cell and Molecular Biology, Center for Biotechnology, Federal University of Rio Grande do Sul (UFRGS) Porto Alegre RS Brazil

## Abstract

*Eragrostis teff* is an Ethiopian native grass plant (*Poaceae* or *Gramineae* family) whose importance as a crop grain has increased in recent years. The aim of this study is to analyze the nutritional composition of its seeds and the mutagenic/antimutagenic activity of the hydroalcoholic extract of the seed flour. Chemical elements (colloquially known as minerals) were determined using Particle-Induced X-ray Emission (PIXE) and Flame Atomic Absorption Spectroscopy (FAAS), while the content of amino acids (aminogram) and fatty acids (profile of fatty acids) were quantified by HPLC. Mutagenic activities were tested using *Salmonella*/microsome assay. Mutagens doxorubicin, 4-nitroquinolin *N*-oxide, methylmethanosulphonate, and aflatoxin B-1 were used in *Salmonella typhimurium* TA98 and TA100 strains to assess antimutagenic activities. The major elements observed were K, P, S, Mg, and Ca. Almost all essential amino acids were observed and the predominance of unsaturated fatty acids in the total oil content of 2.72% (w/w) is also noted, including the two essential fatty acids alpha-linolenic acid (an omega-3 fatty acid) and linoleic acid (an omega-6 fatty acid). Hydroalcoholic extract of *E. teff* seed flour showed antimutagenic activity, protecting against frameshift and base pair substitution mutations. These findings provide valuable information for further development of healthier foods that can be produced with increasing yields and minimal environmental impact.

## Abstract 2

*Eragrostis teff* is an Ethiopian native grass plant (*Poaceae* or *Gramineae* family) whose importance as a crop grain has increased in recent years.

## Graphical abstract

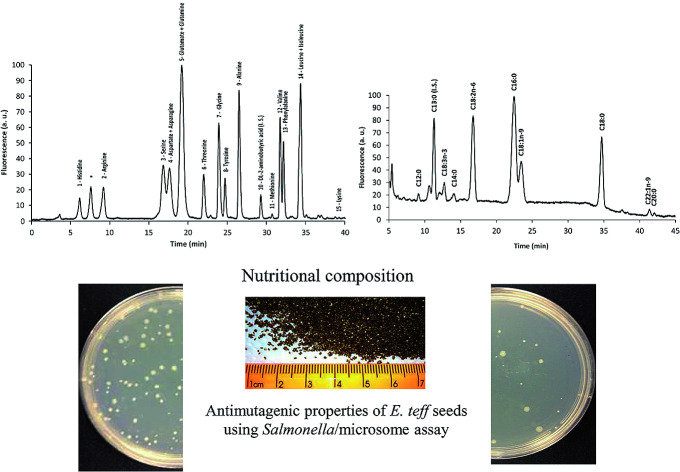

## 1 Introduction

Cells are continuously challenged by DNA damage from several exogenous environmental factors such as ionizing radiation and xenobiotic chemical agents or from endogenous sources like cell metabolic (sub)products. Specific DNA damages may induce mutations that lead to cancer or other diseases and contribute to the aging process. Food with a balanced nutritional composition of amino acids, fatty acids, vitamins and minerals is highly recommended since all cells require these substances in appropriate amounts to maintain their homeostasis.^1,2^ Foods containing the so called chemopreventive agents have the potential to increase life quality and expectancy. They act by means of their antioxidant (mainly anti-free-radical) activities, inhibition of mutagenic agents, by the promotion of detoxifying enzymes and/or providing protection against many oncogenic substances.^3^

*Eragrostis teff* (Zucc.) Trotter belongs to the *Poaceae* family and is an important native staple crop in Ethiopia and Eritrea, where its seeds are used as food (injera and kitta) and to produce beverages (tella). They are revised by Gebremariam *et al.* (2014).^4^ Since this plant is adapted to grow in diverse environmental conditions, it has been cultivated in countries like India, Australia, the United States of America and, more recently, in Paraguay and Brazil. Studies on the nutritional properties of *E. teff* grains have shown high levels of proteins, comparable to barley, wheat, maize and pearl millet, and higher than rye, brown rice and sorghum.^4^ The fat content of *E. teff* grain is higher than that of wheat, rye, and brown rice but lower than that of barley, maize, sorghum, and pearl mille.^4^ Besides the known fact *E. teff* grain is gluten free,^5^ it is also rich in unsaturated fatty acids,^6,7^ and has high levels of K, P, Mg, Ca, Na, Zn, and Fe,^8^ increasing the interest in developing food products such as beverages, breads and pastas from it seed flour.^9^

Considering the expansion of *E. teff* cultivation and the lack of better information about its biological effects associated to its promising nutritional value, the aim of this study was to evaluate the mineral, amino acid, and fatty acid composition of *E. teff* seeds and the mutagenic/antimutagenic effects of hydroalcoholic extract obtained from seed flour. The mutagenic activity is an important aspect to be evaluated in food safety. Antimutagenicity is a desired property in foods as it mitigates genomic instability. To our known this is the first study evaluating the mutagenic/antimutagenic effects of *E. teff* seeds.

## 2 Material and methods

### 2.1 Plant material

The brown type *E. teff* was planted and the seeds harvested on May 2015 by the El Campo farm located in the municipality of Pedro Juan Caballero, Paraguay (22 19′54.41′′S, 55 52′ 22.35′′W; 662 m above sea level). A 50 kg bag of the seeds were donated for the present study and the quartering technique was used to reduce the sample size to about 2 kg without any systematic bias. The seeds were brown with a copper shade, dense (1 g mL^−1^) and hard. Contamination by weed seed was extremely low, nevertheless, it was carefully examined to avoid any contaminant.

About 1 g of this sample, which was also produced by the quartering technique, was then planted again to produce the samples for the exsiccate. Voucher specimens were identified by one of the authors (TBLK) and the exsiccate has been deposited with number ICN 199247 at the Herbarium ICN of the Federal University of Rio Grande do Sul, Porto Alegre, RS, Brazil.

### 2.2 *E. teff* seed and flour preparation

The remaining seeds of the above 2 kg sample were then dried in an oven at 60 °C using layers of 0.5 cm of sample in glass trays following the 012/IV method of the IAL (Adolfo Lutz Institute). They were weighed each twelve hours until a constant weight was observed. This took forty-eight hours to happen and the average loss of weight was 10.25%. This process gave the seeds a noticeable darker shade if compared to the fresh seeds. The seeds were then stored in a hermetically closed bowl until analysis. Prior to each analysis the seed flour was produced using a coffee grinder (Cadence, Brazil) until a thin flour was obtained. This was made in a standard manner: 70 g (approx. 60 mL) was used on each batch and the sample was ground for 2 min.

### 2.3 Analysis of inorganic elements in *E. teff* seed flour

The elemental composition of the *E. teff* seed flour was determined using the Particle-Induced X-ray Emission (PIXE) technique and the Flame Atomic Absorption Spectroscopy (FAAS) using the Adolfo Lutz Institute method 210/IV:2008. Briefly, for the PIXE analysis, *E. teff* seed flour was pressed into thick pellets and placed in the target holder inside the ion beam reaction chamber, which was maintained at a pressure of approximately 10^−6^ mbar. A 3 MV Tandetron accelerator was used to irradiate the target with a 2.0 MeV proton beam and an average current of 3.5 nA. The X-rays derived from samples were detected using a Si (Li) detector with an energy resolution of approximately 150 eV at 5.9 keV. The PIXE spectra were fitted and quantified using the GUPIXWIN software package developed at the University of Guelph (Guelph, Canada)^10^ and the results were expressed in mg/100 g. The analysis procedure followed the standardized protocol described by Johansson *et al.* (1995).^11^ As for the FAAS analysis, the *E. teff* seed flour was first calcinated at 800 °C and then dissolved in acid and subjected to analysis according to the method 210/IV:2008 of the Adolfo Lutz Institute.^12^

### 2.4 Analysis of organic substances in *E. teff* seed flour

#### 2.4.1 Hardware used

An HPLC 525 Instrument (Biotech, Germany) equipped with a thermostated column compartment was employed to separate amino acids and fatty acids. The chromatographic separation was performed on a Hi-Chrom C18 column (250 × 4.6 mm i. d. packed with 5 μm particles) from Hi-Chrom (United Kingdom) and fluorescence detection was used by a laser-induced fluorescence detector of a capillary electrophoresis system PNA8C (a donation of ISB, Brazil). In this, the excitation is induced by a 405 nm diode laser and the detection of the fluorescent light is made by a sensitive CCD camera. Peak areas were calculated (integration of the chromatograms) using the Chromophoreasy software.^13^ The hybridization oven used for the enzymatic hydrolysis of the triacylglycerol was purchased from Amersham Pharmacia Biotech (United Kingdom).

#### 2.4.2 Reagents

The following reagents were purchased from Sigma (St. Louis, USA): trifluoroacetic acid (TFA, HPLC grade), acetonitrile (HPLC grade), 12 N hydrochloric acid (HCL), naphthalene-2,3-dicarboxyaldehyde (NDA), potassium cyanide (KCN), potassium hydroxide (KOH), dl-2-aminobutyric acid (internal standard for the amino acid analysis), amino acid standards, boric acid, methanol (HPLC grade), 3-[4-(bromomethyl)phenyl]-7-(diethylamino)coumarin (MPAC-Br), 18-crown-6 ether, potassium bicarbonate (KHCO_3_), tris(hydroxymethyl)aminomethane (TRIS), acetic acid, *n*-hexane, fatty acid standards including octanoic acid (C8:0), decanoic acid (C10:0), dodecanoic acid (C12:0), tridecanoic acid (C13:0 – internal standard for the fatty acid analysis), myristic acid (C14:0), palmitic acid (C16:0), stearic acid (C18:0), oleic acid (C18:1) linoleic acid (C18:2), linolenic acid (C18:3), arachidic acid (C20:0) and erucic acid (C22:1). The enzyme Lipozyme TL IM was kindly provided by Novozymes Latin America (Araucária, PR, Brazil). Cellulose cartridges 33 × 80 mm from the Unifil brand (Brazil) were purchased from LAS (Porto Alegre, Brazil).

#### 2.4.3 Amino acid analysis

Fractions of 1 g of the *E. teff* seed flour were placed into 50 mL hydrolysis flasks to which 9 mL of 6 N HCl and 2 mL of 10 mM internal standard (dl-2-aminobutyric acid) were added. Approximately 0.2 g of phenol was also added to prevent the oxidation of some amino acids. Flasks were then sealed Teflon and rubber septa, inert using vacuum and ultrapure nitrogen and placed in an oven at 110 °C for 24 h for the hydrolysis of proteins, yielding free amino acids.

After 24 h, the hydrolysis flasks were removed from the oven and cooled. The hydrolyzate was filtered through filter paper. A volume of 5 mL of the filtrate was placed in 50 mL Becker and titrated with 12 N KOH to pH 9. The final volume was adjusted to 20 mL with distilled water.

The derivatization procedure used for the fluorescence detection and quantification of amino acids was modified from Siri *et al.* (2006).^14^ In 500 μL reaction tubes, the following reagents were added in this order: 3 μL of sample; 237 μL of 100 mM borate buffer pH 9; 30 μL of 10 mM KCN pH 9; 30 μL of 20 mM NDA in acetonitrile. The derivatization reaction was left to occur at room temperature (24 °C) for 20 min. After this time, a fraction of derivatized solution was diluted five times with acetonitrile and a 10 μL volume was injected in the HPLC 525 Instrument.

The chromatographic conditions employed were as follows: column temperature was adjusted to 40 °C. The flow rate of solvents was set to 1.0 mL min^−1^ and the mobile phases were acidified water with TFA, pH 2 (mobile phase A) and acetonitrile (mobile phase B). The gradient programming was as follows: 0–12 min, 30% B; 12–14 min, 30–35% B; 14–40 min, 35–80% B; 40–41 min, 80–30% B.

#### 2.4.4 Fatty acid analysis

The oil from flour samples (see Section 2.2) was extracted using the Soxhlet method with *n*-hexane at 60 °C. The system uses two condensers in series: the bottom condenser operates at 60 °C using mineral oil circulation from a thermal bath, and the top condenser uses tap water to condense the remaining hexane vapor. After extraction, the crude oil was subjected to enzymatic hydrolysis using the enzyme Lipozyme TL IM (immobilized on micron size particles, with activity – interesterification unit, IUN – of 250 IUN g^−1^) to obtain free fatty acids from triglycerides.^15^ In the reaction flasks, oil, water, and enzyme were added with the proportions of 20 : 4 : 1, respectively. The mixture was kept in an oven at 45 °C with shaker for 24 h. After hydrolysis, the content was transferred to a 50 mL conical plastic tube and centrifuged at 2000*g* for 10 min to get a clean fatty acid supernatant. A fraction of the upper phase was collected and diluted in methanol to a final concentration of 1 mg mL^−1^ for derivatization.

The derivatization method was modified from Takechi *et al.* (1996)^16^ and is detailed described by Rodrigues *et al.* (2018).^17^ In short, the hydrolyzed samples were mixed in the derivatization reaction flasks and prepared as follows: 5 mg of KHCO_3_, 175 μL propylene carbonate, 15 μL of sample in methanol, 10 μL of 250 μM C13:0 in methanol (internal standard); 18 μL of 10 mM 18-crown-6 in acetonitrile, 18 μL of 5 mM MPAC-Br in acetonitrile, and 14 μL acetonitrile. The mixed solution was warmed to 75 °C and kept at this temperature for 40 min in an mineral oil bath and continuously homogenized using a magnetic stirrer. After this time, the mixture was centrifuged at 1000*g* for 10 min and a 25 μL aliquot of the clean supernatant was injected into the HPLC 525 instrument for analysis. The same procedure was used with the standards of fatty acids and long chain fatty acids dissolved in methanol to obtain the calibration curves. The chromatographic conditions were as follows: the temperature of the column oven was adjusted to 30 °C. The flow rate was set to 1.5 mL min^−1^ and the mobile phases were Tris–acetate/methanol, pH 7.5 (10 : 90, v/v, mobile phase A) and acetonitrile (mobile phase B). The gradient programming was as follows: 0–20 min, 100% A; 20–35 min, 50% A to 50% B; 35–45 min, 100% B.

### 2.5 Preparation of hydroalcoholic extract from *E. teff* seeds

*E. teff* seed flour (125 g) was submitted to maceration using a hydroethanolic solution (ethanol–water, 70 : 30, v/v) at seed flour : hydroethanolic solution (1 : 5 w/v). The solution was filtered through Whatman no 1 filter paper and seed flour was extracted again with the same volume of the hydroethanolic solution. This procedure was repeated five times in consecutive days. After that, the hydroethanolic solutions were pooled and evaporated in a rotary evaporator at 45 °C until the complete drying of the sample. The hydroethanolic extract was then frozen and concentrated by lyophilization to obtain a final yield of 8.21 g (6.84%, w/w) of hydroalcoholic extract of *E. teff* seed flour (HA-Et). This extract was used in mutagenic and antimutagenic assays.

### 2.6 *Salmonella*/microsome mutagenicity assay

Mutagenicity was evaluated using the pre-incubation procedure as reviewed in the previous study of Mortelmans and Zeiger (2000).^18^ Five *Salmonella typhimurium* strains provided by MOLTOX® (Molecular Toxicology Inc., USA) were used. *S. typhimurium* TA1535 and the corresponding isogenic strain TA100 were employed to detect base pair substitutions (DNA target leucine codon [GAG] by proline codon [GGG]); *S. typhimurium* TA98 (DNA target –C–G–C–G–C–G–C–G–; −1) and *S. typhimurium* TA97a (DNA target –C–C–C–C–C–C–; +1 cytosine codon) were employed to detect frameshift mutations; and *S. typhimurium* TA102 was employed to detect transversions or transitions (TAA ochre) since it is sensitive to oxidative, crosslinking, and alkylating mutagens. Briefly, 100 μL of test bacterial cultures (1–2 × 10^9^ cells per mL) were incubated at 37 °C with different amounts of HA-Et in the presence or absence of S9 mix for 20 min, without shaking. Subsequently, 2 mL of soft agar (0.6% agar, 0.5% NaCl, 50 μM histidine, 50 μM biotin, pH 7.4, 42 °C) were added to the test tube and poured immediately onto a plate of minimal agar (1.5% agar, Vogel–Bonner E medium containing 2% glucose). Aflatoxin B1 (AFB-1, 1 μg per plate) was used as positive control for all strains in the presence of metabolic activation (with S9 mix). In the absence of metabolic activation, 4-nitroquinoline *N*-oxide (4-NQO, 0.5 μg per plate) was used for *S. typhimurium* TA98, TA97a, and TA102 strains and sodium azide (NaN_3_, 1 μg per plate) was employed for *S. typhimurium* TA100 and TA1535 strains. Plates were incubated in the dark at 37 °C for 48 h before counting revertant colonies. Assays were repeated twice and the plating for each dose was in triplicate.

### 2.7 *Salmonella*/microsome antimutagenicity assay

*S. typhimurium* TA98 and TA100 were used to assess HA-Et antimutagenicity in co- and pre-treatment procedures. Doxorubicin (DOX) and 4-NQO were used to induce mutations in *S. typhimurium* TA98 without S9 mix. Methylmethanesulphonate (MMS) and DOX were used with *S. typhimurium* TA100 without S9 mix. Aflatoxin-B1 was used with both *S. typhimurium* strains in assays in the presence of S9 mix. In the pre-incubation procedure, HA-Et was incubated with the cultures at 37 °C without shaking, in the presence or absence of S9 mix, for 20 min. A mutagen was then added and the mixture was further incubated at 37 °C for 20 min followed by plating.^19^ In the co-treatment, HA-Et and the mutagen were simultaneously incubated with bacterial cultures at 37 °C without shaking and in the presence or absence of S9 mix, for 20 min followed by plating. All plates were incubated at 37 °C for 48 h before counting revertant colonies. Assays were repeated twice and the plating for each dose was in triplicate.

### 2.8 Data analysis

Results of mutagenic and antimutagenic evaluations were expressed as means ± S.D. and the statistical significance was determined by One-Way Analysis of Variance (ANOVA) complemented by Dunnett's test. In all comparisons, *p* < 0.05 was considered as indicating statistical significance. A test substance was considered mutagenic in the *Salmonella*/microsome assay when significant ANOVA variance was observed, and the mean number of revertants on test plates was at least twice as high as that observed in the negative control plates (or at least three times higher, for the *S. typhimurium* TA1535 strain).

A test substance was considered antimutagenic when a significant decrease in the mean number of revertants was observed on plates containing the test substance plus mutagen in comparison to plates containing only the mutagen. The percentage of inhibition of mutagenicity was calculated as follows: % inhibition = [1 − (*B*/*A*)] × 100, where *A* represents the number of revertants on the plate containing mutagen only, and *B* represents the number of revertants on the plate containing mutagen and antimutagen. The number of spontaneous revertants on the negative control plate was subtracted from each of *A* and *B*. The antimutagenic effect was considered moderate when the inhibitory effect was between 25–40% and strong when the inhibitory effect was higher than 45%. Inhibitory effects of less than 25% were considered weak.

## 3 Results

In order to contribute with specific information about the nutritional value of *E. teff* seeds, we evaluated the composition of seed flour chemical elements (minerals), amino acids, and fatty acids. As shown in Table 1, the most prominent inorganic elements found in *E. teff* seeds were: potassium (K) 638 mg/100 g (PIXE) and 594 mg/100 g (FAAS), sulfur (S) 322 mg/100 g (PIXE), and phosphorous (P) 421 mg/100 g (PIXE). In decreasing order of concentration, we were also able to demonstrate considerable quantities of calcium (Ca) and magnesium (Mg) with more than 100 mg/100 g in each average. In quantities below 100 mg/100 g, we also detected chloride (Cl), silicon (Si), iron (Fe), manganese (Mn), aluminum (Al), zinc (Zn), titanium (Ti), copper (Cu), and bromine (Br). These results were compared with the results obtained in previous studies.^7,20,21^

**Table 1:** Chemical inorganic elements in *E. teff* seeds ranked by average concentration in mg/100 g

	This work	This work	Bultosa and Taylor 2004	El-Alfy *et al.* 2012^c^	Hager *et al.* 2012	Average
Variety	Brown	Brown	Not ment.	Not ment.	Not ment.	
Sample	Dry seeds	Dry seeds	Not ment.	Dry seeds	Fresh seeds	
Method	PIXE	FAAS	Not ment.	SEM-EDX^d^	ICP/AES^e^	
Units	mg/100 g	mg/100 g	mg/100 g	mg/100 g	mg/100 g	mg/100 g
K	638 ± 146	594 ± 6^a^	380	1921.3	382.77 ± 0.45	783
S	322 ± 19			609.0		465
P	421 ± 52		425.4	52.78	361.70 ± 1.10	315
Ca	213 ± 26	71.9 ± 6	165.2	571.1	154.30 ± 0.20	235
Mg	311 ± 62	188 ± 6	181	47.44	168.97 ± 1.45	179
Cl	52.7 ± 4.0			171.19	48.10 ± 3.91	91
Si	70.7 ± 13.8					70.7
Fe	31.5 ± 6.9	24.3 ± 0.6	15.7	25.50	8.53 ± 0.20	21
Na			15.9	59.30	5.98 ± 0.21	27
Mn	10.3 ± 1.4		3.8		3.45 ± 0.04	5.9
Al		5.8 ± 1.9				5.8
Zn	4.72 ± 0.79	4.5 ± 1.9	4.8		4.15 ± 0.01	4.5
Ti	3.99 ± 1.18					4.0
Cu	1.40 ± 0.50	<0.5	2.6		0.93 ± 0.01	1.6
Br	1.39 ± 0.11					1.4
Ni		<0.1				<0.1
Se		<0.1^b^				<0.1
Co		<0.1				<0.1

a Analysis by flame atomic emission spectroscopy instead of FAAS.

b Analysis by FAAS with the aid of a hydride generator.

c These numbers (mg of each element/100 g of dry seeds) were calculated using the factor 59.3. El-Alfy *et al.* (2012) expressed their results of element concentration as % (w/w) in the ashes, which were calculated considering that dry seeds yielded 5.93% ash (or 5.93 g ash/100 g of dry seeds).

d SEM-EDX = Scanning Electron Microscopy with Energy-Dispersive X-ray Spectroscopy.

e ICP/AES, Inductively Coupled Plasma/Atomic Emission Spectroscopy. Method EN ISO 11885 E22.

**Fig. 1:**
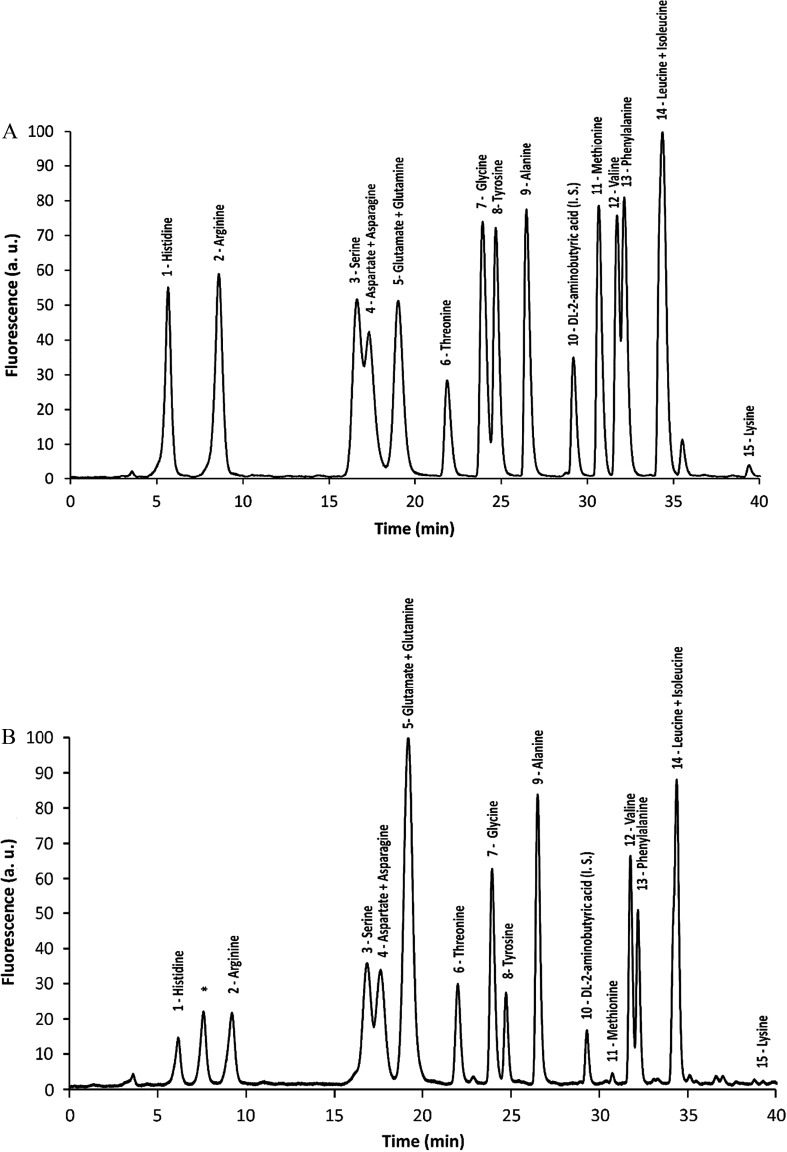
Chromatogram of fluorescent derivatives formed by the reaction between free amino acids with NDA. Standard of amino acids (A) and a sample (B) from *E. teff* seed hydrolyzed proteins. Numbers above chomatographic peaks are referred to the following amino acids: (1) histidine; (2) arginine; (3) serine; (4) aspartic acid/asparagine; (5) glutamic acid/glutamine; (6) threonine; (7) glycine; (8) tyrosine; (9) alanine; (10) dl-2-aminobutyric acid (internal standard); (11) methionine; (12) valine; (13) phenylalanine; (14 + 15) leucine/isoleucine; (16) lysine.

To determine the amino acid composition in *E. teff* seeds, 1 g samples of seed flour were submitted to protein hydrolysis. The resulting free amino acids were derivatized with NDA to yield fluorescent derivatives that were further separated by HPLC and detected. As shown in Table 2 and Fig. 1A (standard) and Fig. 1B (sample), the most abundant amino acids found in *E. teff* seed flour were glutamic acid/glutamine (3.88 g/100 g), leucine/isoleucine (2.29 g/100 g), threonine (1.41 g/100 g), valine (1.09 g/100 g), alanine (1.04 g/100 g), phenylalanine (0.99 g/100 g), serine (0.93 g/100 g), lysine (0.87 g/100 g) and arginine (0.80 g/100 g). In lesser but still appreciable amounts were also detected tyrosine (0.70 g/100 g), glycine (0.68 g/100 g), histidine (0.51 g/100 g), aspartic acid/asparagine (1.39 g/100 g), and methionine (0.06 g/100 g). We were not able to analyze only three of the twenty proteinogenic amino acids due to a limitation of our method: tryptophan (which is partially degraded by acid hydrolysis), cysteine (which forms cystine and this NDA derivative is subjected to fluorescence quenching), and proline (the secondary amine does not react with NDA). These results were also compared with that one obtained by El-Alfy *et al.* (2012).^21^

**Table 2:** Amino acids content in *E. teff* seeds ranked by average concentration in g/100 g

	Present work	El-Alfy *et al.* 2012	Average
Variety	Brown	Red	
Sample	Dry seeds	Dry seeds	
Method	HPLC	Not ment.	
Units	g/100 g	g/100 g	g/100 g
Glutamate + glutamine	3.88	3.86	3.87
Leucine + isoleucine	2.29	2.67	2.48
Aspartate + asparagine	1.39	2.17	1.78
Tryptophan	ND	ND	1.30
Proline	ND	1.28	1.28
Arginine	0.80	1.66	1.23
Threonine	1.41	1.01	1.21
Lysine	0.87	1.35	1.11
Valine	1.09	1.11	1.10
Glycine	0.68	1.44	1.06
Alanine	1.04	1.03	1.04
Serine	0.93	1.02	0.98
Phenylalanine	0.99	0.85	0.92
Tyrosine	0.70	ND	0.70
Histidine	0.51	0.72	0.62
Cystine	ND	0.45	0.45
Methinonine	0.06	0.44	0.25

**Fig. 2:**
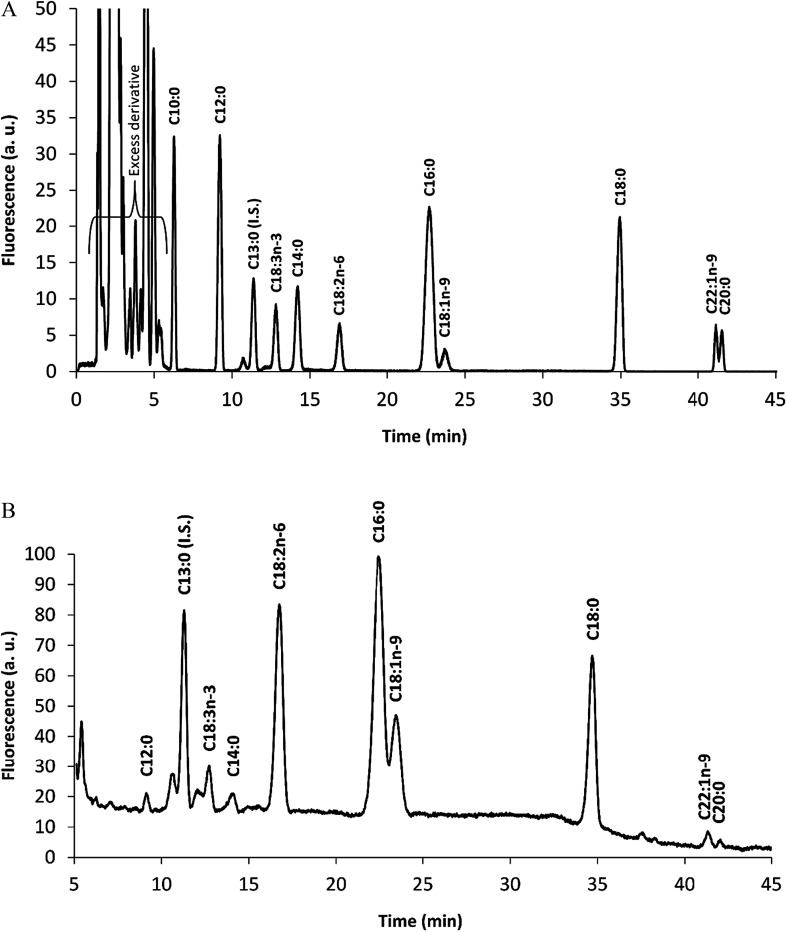
Chromatogram of fatty acid-MPAC-Br derivatives. Standard of fatty acids (A) and a sample (B). Samples of *E. teff* seed flour had the oils extracted and enzymatically hydrolyzed to deliver the fatty acids. These fatty acids were derivatized with MPAC-Br. The following fatty acids were found in the sample: C13:0, tridecanoic acid (internal standard, I.S.); C18:3, linolenic acid; C18:2, linoleic acid; C16:0, palmitic acid; C18:1, oleic acid; C18:0, stearic acid. Detection was performed with LIF at 405 nm.

The hexane soluble compounds of *E. teff* seed four was 2.72 g/100 g according to the Soxhlet method operated at 60 °C used in this work. This oil was then hydrolyzed using an enzymatic procedure (Section 2.4.4) and the total fatty acids were derivatized with MPAC-Br and separated by HPLC and quantified by fluorescence detection. As shown in Table 3 and Fig. 2A (standard) and Fig. 2B (sample), more than 70% of *E. teff* seed oil is composed by linoleic acid (C18:2, 33.42%) and oleic acid (C18:1, 27.53%), which are important unsaturated fatty acids. Palmitic acid (C16:0, 14.91%), stearic acid (C18:0, 12.21%), and linolenic acid (C18:3, 5.97%) were the most abundant fatty acids in the oil. These results were compared with other two studies,^7,21^ and the data were ranked by the average value of the three results mentioned in Table 3. The total oil content found was 2.72 g/100 g and this was also compared with the literature^7,20^ resulting in an average of 3.20 g/100 g (Table 4).

**Table 3:** Fatty acids content in *E. teff* seeds ranked by average profiles (%)

	This work	El-Alfy *et al.* 2012	Hager *et al.* 2012	Averages
Variety	Brown	Not ment.	Not ment.	
Sample	Dry seeds	Not ment.	Fresh seeds	
Method	HPLC	GC	GC	
Units	Profile (%)	Profile (%)	Profile (%)	Profile (%)
Linoleic – C18:2	33.42	12.94	49.99	35.75
Oleic – C18:1	27.53	32.41	29.47	29.80
Palmitic – C16:0	14.91	14.52	10.86	13.43
α-Linolenic – C18:3	5.97	23.83	2.29	7.07
Stearic – C18:0	12.21	4.20	4.14	6.85
Arachidonic – C20:4	ND	3.14	ND	3.14
Erucic – C22:1	1.82	ND	ND	1.82
Arachidic – C20:0	1.74	ND	ND	1.74
Myristic – C14:0	1.27	0.47	0.22	0.65
Lauric – C12:0	1.13	0.17	ND	0.65
Butyric – C4:0	ND	0.32	ND	0.32
Lauric – C12:0	ND	0.17	ND	0.17
Palmitoleic – C16:1	ND	0.14	ND	0.14
Caprylyc – C8:0	ND	0.12	ND	0.12
Caproic – C6:0	ND	0.13	ND	0.13
Capric – C10:0	<0.04	0.08	ND	0.08
Other fatty acids	ND	3.85	0.78	2.32
Total	100	92.47	97.75	

**Table 4:** Fatty acids content in *E. teff* seeds ranked by average profile (%)

	This work	Hager *et al.* 2012	Bultosa and Taylor 2004	Average
Variety	Brown	Not ment.	Not ment.	
Sample	Dry seeds	Fresh seeds	Fresh seeds	
Method	Soxhlet (hexan)	AACCI method 30-10.01	Soxhlet	
Units	g/100 g	g/100 g	5/100 g	g/100 g
Oil content	2.72	4.39	2.5	3.20

Considering the increasing interest in *E. teff* derived foods and beverages, we also assessed its possible mutagenic or antimutagenic effects. To do so, hydroalcoholic extracts (Section 2.5) obtained from *E. teff* seed flour (HA-Et) were tested by *Salmonella*/microsome assay. As shown in Table 5, HA-Et was not able to induce mutations in the strains of *S. typhimurium* TA98, TA97a, TA100, TA1535, or TA102, neither in the absence nor in the presence of S9 mix. In fact, HA-Et increased significantly the revertant numbers of colonies of *S. typhimurium* TA102 in the absence of S9 mix, however without reaching an MI ≥ 2.0, indicating a negative result of mutagenicity. Similarly, there was a significant increase in *S. typhimurium* TA1535 revertant colonies at a dose of 5000 μg per plate of HA-Et in the presence of S9 mix. Nevertheless, MI did not reach values higher than three to HA-Et be considered a positive mutagen to this strain.

**Table 5:** Induction of his + revertants in *S. typhimurium* strains by *E. teff* seed hydroalcoholic extracts with and without metabolic activation

Substance	Concentration (μg per plate)	TA98^a^, rev/plate	MI^b^	TA97a^a^, rev/plate	MI^b^	TA100^a^, rev/plate	MI^b^	TA1535^a^, rev/plate	MI^b^	TA102^a^, rev/plate	MI^b^
**Without metabolic activation (−S9)**											
NC^c^	—	32.7 ± 3.1	—	106.3 ± 3.5	—	106.0 ± 1.0	—	9.0 ± 4.0	—	394.7 ± 19.4	—
*E. teff*	250	21.3 ± 1.5	0.65	96.7 ± 12.5	0.91	97.7 ± 19.0	0.92	10.0 ± 1.7	1.11	421.7 ± 80.9	1.06
	500	23.0 ± 2.6	0.70	73.7 ± 5.5	0.69	101.3 ± 26.2	0.95	13.3 ± 2.9	1.48	424.3 ± 23.5	1.07
	1000	24.7 ± 8.0	0.75	84.7 ± 26.3	0.79	106.7 ± 17.8	1.00	14.3 ± 1.5	1.59	541.7 ± 58.1*	1.37
	2000	23.7 ± 1.5	0.72	69.3 ± 11.7	0.65	102.0 ± 3.6	0.96	15.3 ± 1.5	1.70	601.3 ± 43.6**	1.52
	5000	26.0 ± 5.6	0.79	71.0 ± 18.3	0.67	111.3 ± 10.8	1.05	16.3 ± 6.7	1.81	671.3 ± 74.3***	1.70
PC^d^	0.5 (4NQO), 1.0 (NaN_3_)	185.7 ± 25.0***	5.68	278.0 ± 57.7***	2.61	1084.0 ± 94.5***	10.22	505.0 ± 38.0***	56.10	4658.0 ± 584.0***	11.80

**With metabolic activation (+S9)**											
NC^c^	—	29.7 ± 2.1	—	99.2 ± 17.8	—	107.7 ± 18.9	—	12.7 ± 5.1	—	452.6 ± 29.2	—
*E. teff*	250	26.0 ± 7.6	0.88	77.7 ± 11.6	0.78	98.0 ± 11.4	0.91	10.7 ± 3.2	0.84	472.4 ± 33.5	1.04
	500	23.3 ± 5.1	0.78	96.7 ± 4.0	0.97	103.7 ± 10.3	0.96	14.0 ± 2.6	1.10	408.8 ± 32.7	0.90
	1000	26.3 ± 5.5	0.89	109.0 ± 2.6	1.10	109.3 ± 23.1	1.01	9.7 ± 4.0	0.76	405.2 ± 93.0	0.90
	2000	27.3 ± 5.1	0.92	94.7 ± 15.1	0.95	112.0 ± 9.2	1.04	19.7 ± 7.1	1.55	428.4 ± 80.1	0.95
	5000	36.3 ± 11.1	1.22	109.3 ± 9.5	1.10	115.0 ± 12.1	1.07	27.3 ± 4.1*	2.15	474.4 ± 40.8	1.05
PC^d^	1.0 (AFB-1)	571.0 ± 48.1***	19.23	270.0 ± 7.1***	2.72	1206.0 ± 118.1***	11.20	70.0 ± 11.3 ***	5.51	1466.0 ± 55.9***	3.24

a Number of revertants/plate: mean ± SD.

b MI: mutagenic index (no of his + induced in the sample/no of spontaneous his + in the negative control).

c NC: negative control (70% dimethylsulfoxide in distillated water, 10 μL, used as a solvent of the extract).

d PC: positive control: (−S9) NaN_3_ (sodium azide) to TA100 and TA1535; 4-NQO (4-nitroquinoline *N*-oxide) to TA97a, TA98 and TA102; (+S9) AFB-1 (aflatoxin -B1); significantly different in relation to the negative control. *p < 0.05; **p < 0.01; ***p < 0.001 (ANOVA, Dunnett's test).

Interestingly, the HA-Et was able to decrease the mutagenicity induced by DOX and 4-NQO on *S. typhimurium* TA98 when a co-treatment was performed in the absence of S9 mix (Table 6). With *S. typhimurium* TA100, HA-Et also decreased the mutagenicity effects of DOX and MMS when tested in pre-treatment (Table 7). In the presence of S9 mix, the extract was able to reduce the mutagenicity of aflatoxin B1 on both *S. typhimurium* strains mainly in pre-treatments (Table 8).

**Table 6:** Antimutagenicity of *E. teff* seed hydroalcoholic extracts on *S. typhimurium* TA98 strain in the absence of S9 mix

	HA-Et concentrations (μg per plate)	Revertants/plate (mean ± SD)	Revertants/plate^d^ (mean ± SD) (I%)
Pre-treatment	—	NC^a^	NC
	0	25.7 ± 8.1	26.7 ± 2.1
	—	DOX^b^	4-NQO^c^
	0	189.3± 39.4	273.3 ± 23.9
	250	155.7 ± 32.8	258.7 ± 32.6
	500	175.5 ± 69.9	282.3 ± 56.7
	1000	154.5 ± 87.1	247.0 ± 77.2
	2000	128.5 ± 52.1	337.0 ± 11.3
	5000	173.3 ± 98.1	267.0 ± 15.6
Co-treatment	—	NC	NC
	0	29.0 ± 4.6	25.0 ± 4.5
	—	DOX	4-NQO
	0	388.0 ± 18.4	337.2 ± 30.6
	250	233.7 ± 94.2	316.0 ± 41.0
	500	477.3 ± 70.0	287.7 ± 16.3
	1000	344.3 ± 21.8	165.7 ± 70.7 ** (54.9)
	2000	345.0 ± 17.0	173.3 ± 54.4 ** (52.5)
	5000	252.3 ± 34.6 *(37.8)	157.7 ± 79.0 ** (57.5)

a Negative control: 70% dimethylsulfoxide in distillated water, 10 μL, used as a solvent of the extract.

b Doxorubicin at 1 μg per plate.

c 4-Nitroquinoline *N*-oxide at 0.5 μg per plate.

d Percentage inhibition = [1 − (*B*/*A*)] × 100, where *A* represents the number of revertants on the plate containing mutagen only and *B* represents the number of revertants on the plate containing mutagen and HA-Et. The number of revertants on the NC plate was subtracted from each of *A* and *B*. Significant difference in relation to mutagen: *p < 0.05; **p < 0.01 (ANOVA, Dunnett's test).

**Table 7:** Antimutagenicity of *E. teff* seed hydroalcoholic extract on *S. typhimurium* TA100 strain in the absence of S9 mix

	HA-Et concentrations (μg per plate)	Revertants/plate^d^ (mean ± SD) (I%)	Revertants/plate (mean ± SD) (I%)
Pre-treatment	—	NC^a^	NC
	0	93.7 ± 8.1	115.0 ± 11.8
	—	DOX^b^	MMS^c^
	0	214.3 ± 36.1	353.3 ± 37.9
	250	138.0 ± 6.9** (63.3)	293.0 ± 14.0* (25.3)
	500	96.7 ± 14.3*** (97.5)	273.7 ± 16.1** (33.4)
	1000	128.0 ± 15.6*** (71.6)	287.3 ± 14.9* (27.7)
	2000	140.7 ± 20.7** (61.0)	265.0 ± 27.1** (37.1)
	5000	143.7 ± 9.6** (58.5)	334.0 ± 29.6
Co-treatment	—	NC	NC
	0	99.7 ± 10.7	127.3 ± 16.9
	—	DOX	MMS
	0	209.3 ± 59.7	426.0 ± 15.5
	250	204.8 ± 28.7	463.7 ± 41.7
	500	174.6 ± 38.7	376.7 ± 28.9
	1000	177.2 ± 14.7	388.7 ± 45.8
	2000	197.8 ± 13.7	346.7 ± 21.1* (26.6)
	5000	183.5 ± 21.3	365.3 ± 28.9

a Negative control: 70% dimethylsulfoxide in distillated water, 10 μL, used as a solvent of the extract.

b Doxorubicin at 1 μg per plate.

c Methylmethanesulfonate at 100 μg per plate.

d Percentage inhibition = [1 − (*B*/*A*)] × 100, where *A* represents the number of revertants on the plate containing mutagen only and *B* represents the number of revertants on the plate containing mutagen and HA-Et. The number of revertants on the NC plate was subtracted from each of *A* and *B*. Significant difference in relation to mutagen *p < 0.05; **p < 0.01; ***p < 0.001 (ANOVA, Dunnett's test).

**Table 8:** Antimutagenicity of *E. teff* seed hydroalcoholic extract on *S. typhimurium* TA98 and TA100 strains in the presence of S9 mix

	HA-Et concentrations (μg per plate)	TA98^c^, revertants/plate (mean ± SD) (I%)	TA100, revertants/plate (mean ± SD) (I%)
Pre-treatment	—	NC^a^	NC
	0	25.8 ± 4.9	101.0 ± 1.7
	—	AFB-1^b^	AFB-1
	0	529.0 ± 56.5	686.0 ± 158.0
	250	617.7 ± 42.9	854.3 ± 30.7
	500	459.7 ± 105.0	560.0 ± 150.9
	1000	532.0 ± 97.6	462.7 ± 62.7
	2000	297.3 ± 41.0** (46.1)	507.7 ± 104.7
	5000	184.0 ± 5.7*** (68.6)	303.7 ± 23.6** (65.4)
Co-treatment	—	NC	NC
	0	33.0 ± 3.5	111.8 ± 11.9
	—	AFB-1	AFB-1
	0	670.0 ± 71.1	1116.0 ± 2.1
	250	626.7 ± 19.0	1021.0 ± 30.4
	500	640.3 ± 35.4	862.0 ± 82.3
	1000	627.0 ± 18.4	915.0 ± 67.1
	2000	545.3 ± 121.8	820.3 ± 64.8* (29.5)
	5000	194.3 ± 54.0*** (74.7)	1082.0 ± 136.1

a Negative control: 70% dimethylsulfoxide in distillated water, 10 μL, used as a solvent of the extract.

b Aflatoxin-B1 at 1 μg per plate.

c Percentage inhibition = [1 − (*B*/*A*)] × 100, where *A* represents the number of revertants on the plate containing mutagen only and *B* represents the number of revertants on the plate containing mutagen and HA-Et. The number of revertants on the NC plate was subtracted from each of *A* and *B*. Significant difference in relation to mutagen *p < 0.05; **p < 0.01; ***p < 0.001 (ANOVA, Dunnett's test).

## 4 Discussion

The high nutritional value of *E. teff* grains has strengthened its potential as food supplement to promote and maintain health.^9^ In the present study, chemical elements (minerals), amino acids, and fatty acids of *E. teff* seed flour were quantified in order to check its true richness in such compounds and possible mutagenic/antimutagenic effects were assessed to evaluate its potential risk or chemopreventive capabilities.

The chemical element (mineral) composition of *E. teff* seeds determined by PIXE and/or FAAS showed the presence of K, S, P, Ca, Mg, Fe, Mn, Zn, and Cu as important constituents (Table 1) which are used as cofactors by many enzymes, including DNA repair proteins.^22,23^ All essential amino acids for the human nutrition were found in appreciable amounts in seeds (Table 2 and Fig. 1B), excepting tryptophan which could not be accessed with certainty due to the degradation of the acid hydrolysis step. Glutamic acid/glutamine and threonine were the major amino acids present, followed by leucine/isoleucine and valine. Among fatty acids, the unsaturated linoleic and oleic acids were observed in much higher levels in *E. teff* seed oil than the saturated palmitic and stearic acids (Table 3 and Fig. 2B).

The results of inorganic elements, amino acids, fatty acids, and total oil contents (Tables 1–4) were compared with those obtained in previous studies and showed agreement within the analytical errors and natural variations expected among seeds produced by different soil and cultivation conditions, and under different weather variations.

Hydroalcoholic extract from *E. teff* seeds was shown to be not mutagenic to five *S. typhimurium* strains used in our assays, either in the presence or in the absence of metabolic activation (Table 5). Although the MI value was lower than 2 when assaying *S. typhimurium* TA102, there was a significant dose-dependent increase in the revertant number of colonies for this strain. Interestingly, the mineral composition of *E. teff* seeds revealed a high Fe level which may increase the generation of reactive oxygen species (ROS) by Fenton reaction in the presence of oxygen during HA-Et incubation with this strain which is sensitive to oxidative damages.^18^ In the presence of a metabolic system (S9 mix), no significant mutagenic result was observed.

Notwithstanding, HA-Et extracts exhibited antimutagenic effects on different *S. typhimurium* strains. When *S. typhimurium* TA98 was co-treated with HA-Et and 4-NQO, a significant decrease in the mutagenicity was observed, with I% higher than 45% (Table 6). 4-NQO have shown that it is metabolized into *O*,*O*-diacetyl-4-hydroxy-aminoquinoline 1-oxide (revised in Stankowski *et al.*, 2011).^24^ It forms covalent adducts to C8 or N2 of deoxyguanosine and N6 of deoxyadenosine in DNA. Besides forming monoadducts with purine bases, 4-NQO mutagenic mechanisms are implicated in increasing ROS by undergoing redox cycling and generating superoxide radical and hydrogen peroxide. Additionally, it can react directly with glutathione (GSH), an important tripeptide antioxidant.^24^ Since it is known that 4-NQO reacts with peptides like GSH, it is possible that it may also react with peptides and amino acids present in the extract, decreasing its own availability and thus avoiding the formation of ROS. Therefore, 4-NQO may have its mutagenic effects decreased by HA-Et, making the co-treatment more effective than the pre-treatment.

*E. teff* seeds present high levels of glutamine which is known to exhibit antioxidant effects.^25^ Other plausible reason for the antimutagenic capability of the *E. teff* seed extract could be attributed to the presence of fatty acids. Eicosanoid acid (C20:0) has been shown to decrease the mutagenic activity of 4-NQO on *S. typhimurium* TA98.^26^

DOX, an antibiotic belonging to the anthracycline group and also used in human cancer chemotherapy, induced high mutagenic effects on *S. typhimurium* TA98 (Table 6). DOX is able to intercalate DNA bases, inducing frameshift mutations, besides increasing ROS.^27^ In co-treatment with HA-Et at a dose of 5000 μg per plate, there was a significant decrease in the number of revertant colonies with a moderate inhibition reaching 37.8%. The high levels of linoleic and oleic acids found in *E. teff* seeds (33.42 and 27.53%, respectively), are also known to have antimutagenic effects against dounomycin,^28^ a drug also belonging to the anthracycline group. Thus, the antimutagenic effect observed against DOX may, in this case, be in part by the presence of these specific fatty acids in the *E. teff* seed oil. The intercalation and crosslink induced by DOX, which may increase frameshift mutations, are mainly repaired by the nucleotide excision repair (NER) mechanism.^27^ This type of repair mechanism is absent in *S. typhimurium* TA98 due to the deletion of the *uvrB* gene.^18^ Therefore, the antimutagenic effects observed on this strain may be better explained by direct reactions of HA-Et components with DOX or ROS, which is a more plausible explanation than their effects on repair systems.

DOX also forms DNA adducts and inhibits topoisomerase 2 enzyme (mainly human Top2α and Top2β), impairing replication and transcription.^27^ Bacteria generally contain four topoisomerases (Top I, Top III, gyrase, and Top IV) which are sensitive to other antibiotics.^29^ Although with a weaker effect on *S. typhimurium* TA98, DOX induced mutagenic effect on *S. typhimurium* TA100, likely inducing base pair substitution mutations by the formation of DNA adducts (Table 7). Pre-treatment with HA-Et significantly decreased the mutagenicity induced by DOX, showing a strong antimutagenic effect. On contrary, HA-Et co-treatment did not show a significant decrease of such effects. The presence of amino acids and fatty acids in HA-Et could improve the ability of this strain to repair the DOX-damaged DNA. Amino acids and fatty acids supplied with HA-Et could improve substrate levels and energetic conditions to allow the more efficient production of proteins, including repair enzymes, increasing *S. typhimurium* resistance to DNA damage. In addition, glutamine, one of the amino acids found in high levels in *E. teff*, has shown antimutagenic activities against DOX in mammalian cells.^30^

Thus, HA-Et was more efficient as an antimutagenic agent against DOX in TA100 strain, in pre-treatment procedure, suggesting prevention of base pair substitution mutations induced by DOX. However, HA-Et was not able to decrease the mutagenic effect of DOX in TA98 strain, except in co-treatment at highest concentration, suggesting an antigenotoxic effect related to its antioxidant-like properties, mostly decreasing ROS generated by DOX, not avoiding DOX intercalation which can lead to frameshift mutations.

MMS is a monofunctional alkylating agent that transfers a single alkyl group to DNA and the consequent major adducts formed are N7-methylguanosine (N7meG) and O6- methylguanosine (O6meG).^31^ After pre-treatment of *S. typhimurium* TA100 with HA-Et, the extract significantly decreased the mutagenicity of MMS, with moderate antimutagenic activity (Table 7), suggesting that it blocked, at least in part, the reaction of MMS with DNA, decreasing DNA alkylation or improving DNA repair. Although *S. typhimurium* TA100 is defective in NER due to deletion of *uvrB*,^18^ the base excision repair (BER) mechanism may be assessed to remove DNA adducts including alkylated bases. In addition, DNA alkylation may be repaired by the AlkB dioxygenase enzyme, catalyzing the direct reversal of *N*-alkyl lesions such as 1meA and 3meC, or *via* the O6-methylguanine-DNA methyltransferase (MGMT) repair protein that directly repairs O6meG and O6C1-ethylG lesions.^31^ The previous contact of HA-Et with the bacteria may improve the repair mechanisms to MMS damage. As previously stated, glutamic acid/glutamate were the most abundant amino acids in *E. teff* seed extracts. The amide group of glutamine is essential for purine and pyrimidine *de novo* synthesis and thus its availability may influence the amount of nucleotides produced in a cell and might provide a regulatory mechanism for DNA-repair.^32^ In addition, inorganic elements present in *E. teff* like Fe and Zn may also act as cofactors of repair enzymes.^22,23^ However, the antimutagenic effect of 19 amino acids against the *N*-methyl *N*′-nitro *N*-nitrosoguanidine (MNNG) alkylating agent was determined in a study using *S. typhimurium* TA100 in co-treatment and results showed that the ability to decrease mutagenicity was specific to each amino acid, suggesting the involvement of specific lateral groups with possible direct interaction with MNNG.^33^ A similar mechanism may have occurred between amino acids from *E teff* and MMS, although these two alkylating mutagens have considerably different chemical structures.

It is interesting to note that the antimutagenic activity against DOX in TA100 strain in pre-treatment procedure was stronger at 500 μg per plate (*I*% = 97.5), decreasing in the highest concentrations. When MMS was used, the antimutagenic effect was maintained similar in concentration from 250 to 2000 μg per plate and it was not significant in 5000 μg per plate. This profile of results suggests there is a limiting dose to the antimutagenic effect from which other effects begin to interfere and decrease the antimutagenic activity.

In order to study the antimutagenic effect of HA-Et on pro-mutagens, AFB-1 was used on *S. typhimurium* TA98 and TA100 in the presence of S9 mix. AFB-1 is a mycotoxin often contaminating many food products and one of the most potent naturally occurring mutagens and hepatocarcinogens known.^34^ AFB-1 is metabolized by cytochrome P450 (CYP450) enzymes to its reactive intermediate *exo*-AFB-8,9-epoxide and other oxidized metabolites that form mutagenic adducts. The S9 mix used in *Salmonella*/microsome assay is a metabolic system containing CYP450 enzymes, including CYP1A2 and CYP3A4, the most important CYP450 involved in the inactivation of AFB-1.^34^ Thus, an alteration in the function of the enzymes may result in altered reaction rates and differential pathways of AFB-1 metabolism. As showed in Table 8, the antimutagenic effect was more pronounced when HA-Et was administered in pre-treatment, reaching 68.6% and 65.4% with *S. typhimurium* TA98 and TA100, respectively. In HA-Et co-treatment, only with higher concentrations of the extract the mutations induced by AF-B1 decreased in *S. typhimurium* TA98 (74.7%), while with *S. typhimurium* TA100, there was a significant decrease only at a concentration of 2000 μg per plate (29.4%). Probably the pre-treatment assured previous contact of HA-Et with metabolic enzymes of the S9 mix favoring the inhibition of biotransformation of AFB-1 by modulating S9 mix enzymes needed to activate AFB-1 to its mutagen epoxide derivatives.^34^ However, more studies are needed to confirm this hypothesis. In addition, it cannot be discarded the effects of some HA-Et components that may have reacted directly with AFB-1 or its metabolites, decreasing their concentrations, since there was a strong decrease in mutagenic activity (74.7%) in HA-Et co-treatment in *S. typhimurium* TA98 in higher concentration. Purple and white rice extracts have shown antimutagenic effects on *S. typhimurium* TA98 against AFB-1 and the results were attributed to the suppression of CYP1A2 activity and a direct attack on electrophilic mutagens.^35,36^ SH-containing compounds, including NAC, reduced GSH, *N*-2 mercaptopropionylglycine, and cysteine were able to inactivate the mutagenic activity of AFB-1 in *Salmonella*/microsome assay (revised in Friedman and Rasooly 2013).^37^ It is known that thiols are potent nucleophiles that may competitively inhibit the interaction of the AFB-8,9-epoxide with DNA.^37^ However, in this study, thiol-containing amino acids like cysteine were not found in *E. teff* seed extracts. Other studies have reported the formation of adducts between products of AFB-1 hydrolysis and oxidation with free or protein-bound lysine residues.^38,39^ Lysine was found in *E. teff* seed extracts at a concentration of 0.87 g/100 g sample, which may have contributed to decrease AFB-1 mutagenicity by direct interaction. Previous studies have shown the antimutagenic effects of Mn complexes synthesized with amino acids threonine, serine, tyrosine, glutamine, and asparagine by decreasing the micronucleus frequency induced by AFB-1 on human lymphocyte cultures, likely by binding AFB-8,9-epoxide and leading to its inactivation.^40,41^

The branched-chain amino acids (BCAA) valine, leucine, and isoleucine are known to significantly inhibit the incidence of liver neoplasms in mice.^42,43^ These amino acids were found in high concentrations in *E. teff* seed extracts. The supplementation with BCAA is known to improve protein-energy malnutrition and hypoalbuminemia, resulting in an improvement in the quality of life and in the prognosis of cirrhotic patients.^44^ The antimutagenicity of HA-Et against AFB-1 observed here may be possibly associated with the presence of BCAA in *E. teff* seeds, suggesting a potential hepatoprotection capability.

The combined results showed that HA-Et pre-treatment has an effective antimutagenic effect on *S. typhimurium* TA100 in the presence of either DOX or MMS by probably protecting DNA against adduct production which, in turn, may lead to base pair substitution mutations. When assaying *S. typhimurium* TA98, which allows the detection of frameshift mutations, the co-treatment of HA-Et showed antimutagenic effects against DOX and 4-NQO most likely by exerting antioxidant activities from its organic components, inactivating ROS or directly reacting with the mutagens. In addition, HA-Et was able to decrease the mutagenic effects of AFB-1 on both *S. typhimurium* strains either by interfering with the metabolism of this promutagen or by functioning as a blocking agent.

In conclusion, the group of analysis we conducted allowed us to show and confirm the richness and uniqueness of *E. teff* seeds in amino acids, inorganic elements, and fatty acids. Additionally, *E. teff* seed extracts were shown to be able to reduce or help to repair gene mutations of both categories of frameshift and base pair substitution, acting so possibly by the modulation of xenobiotic metabolizing enzymes and/or by directly reacting with the mutagens. Therefore, *E. teff* seeds may be a rich source of nutritional agents with chemopreventive effects. Further studies on the antimutagenicity of *E. teff* seed extract are still needed to fully elucidate its chemopreventive mechanisms of action.

## Conflicts of interest

The authors declare no conflict of interest.

## Supplementary Material
